# Dataset on schistosomiasis control using potassium usnate against *Biomphalaria glabrata* at different developmental stage and *Schistosoma mansoni* cercariae

**DOI:** 10.1016/j.dib.2018.10.119

**Published:** 2018-10-27

**Authors:** Hallysson Douglas Andrade de Araújo, Ana Maria Mendonça de Albuquerque Melo, Williams Nascimento de Siqueira, Mônica Cristina Barroso Martins, André de Lima Aires, Mônica Camelo Pessoa de Azevedo Albuquerque, Nicácio Henrique da Silva, Vera Lúcia de Menezes Lima

**Affiliations:** a Departamento de Bioquímica, Centro de Biociências, Universidade Federal de Pernambuco, Avenida Prof. Moraes Rego, 1235 Cidade Universitária, CEP 50670-901 Recife, PE, Brazil; b Departamento de Biofísica e Radiobiologia, Centro de Biociências, Universidade Federal de Pernambuco, Avenida Prof. Moraes Rego, 1235 Cidade Universitária, CEP 50670-901 Recife, PE, Brazil; c Departamento de Medicina Tropical, Centro Ciências da Saúde - CCS, Universidade Federal de Pernambuco, Avenida Prof. Moraes Rego, 1235 Cidade Universitária, CEP 50670-901 Recife, PE, Brazil; d Laboratório de Imunologia Keizo Asami - LIKA, Universidade Federal de Pernambuco, Avenida Prof. Moraes Rego, 1235 Cidade Universitária, CEP 50670-901 Recife, PE, Brazil

## Abstract

This text presents complementary data corresponding to schistosomiasis mansoni׳s vector control and toxicity on *Schistosoma mansoni* cercariae using potassium usnate. This information support our research article “Potassium Usnate Toxicity Against Embryonic Stages of the Snail *Biomphalaria glabrata* and *Schistosoma mansoni* Cercariae” [1], and focuses on the analysis of the detailed data regarding the different concentrations of potassium usnate and their efficiency to *B. glabrata* mortality and non-viability and *S. mansoni* cercariae mortality etiologic agent of the disease.

**Specifications table**

**Table:** 

Subject area	*Chemistry, Biology*
More specific subject area	*Natural products biochemistry*
Type of data	*Tables*
How data was acquired	Stereoscopic microscope (Wild M3B, Heerbrugg, Switzerland) and an inverted microscope (Leica DM IL)
Data format	Analyzed
Experimental factors	Usnic acid purification from *Cladonia substellata* lichen and subsequently synthesis of the potassium usnate
Experimental features	Embryonic stages *B. glabrata* unviability and mortality tests and *S. mansoni* cercariae mortality assay over potassium usnate treatments were evaluated
Data source location	*Recife, Brazil*
Data accessibility	Data found in this article
Related research article	H.D.A. Araújo, A.M.M.A. Melo, W.N. Siqueira, M.C.B. Martins, A.L. Aires, M.C.P.A. Albuquerque, N.H. Silva, V.L.M. Lima, Potassium usnate toxicity against embryonic stages of the snail *Biomphalaria glabrata* and *Schistosoma mansoni* cercariae. Acta Tropica [1]

**Value of the data**

• The data detail the embryotoxic activity of potassium usnate on the evolutionary stages (blastula, gastrula, trophorora, veliger, and hippo stage) of *B. glabrata*, correlating the different concentrations applied to the respective stages.
• Data report a more detailed view of the malformations and death, express in LC_10_, LC_50_, and LC_90_, of the different stages of development of *B. glabrata* embryos, of the original article as to their minimum concentration to reach the LC_100_ of the embryos.
• The data of different times (15, 30, 60, 90 and 120 min) allow us to infer possible time intervals to obtain effective results (LC_10_, LC_50_ and LC_90_) on *S. mansoni* cercariae.

## 1 Data

The data presented in this work provide results related to the unviability, (malformations and death) of embryos in the stages of blastula, gastrula, trophorora, veliger, and hippo stage of the *Biomphalaria glabrata* after different treatments with the potassium usnate (Table 1), as well as the potassium usnate activity on cercariae of *Schistosoma mansoni* (Table 2).

**Table 1:** *Biomphalaria glabrata* embryos unviability under different treatments with potassium usnate.

Embryonic stages	Treatment (μg/mL)	Viable embryos (%)	Unviable embryos (%)		
		Complete development	Malformed	Dead	Total unviable
Blastula	Potassium usnate				
	1.0	99.3 ± 0.5	0.3 ± 0.5	0.3 ± 0.5	0.6 (1.0)
	1.5	99.0 ± 1.0	0.3 ± 0.5	1.0 ± 0.0	1.3 (0.5)
	2.0	98.0 ± 0.0	0.6 ± 0.5	2.0 ± 1.0	2.6 (1.5)
	2.5	75.7 ± 10.4	14.0 ± 6.0	10.3 ± 4.5	24.3 (10.5)^a^
	3.0	74.3 ± 5.8	15.3 ± 4.7	10.6 ± 1.5	25.9 (6.2)^c^
	3.5	66.6 ± 3.0	17.6 ± 4.1	15.6 ± 5.1	33.2 (9.2)^c^
	4.0	62.0 ± 6.0	13.0 ± 3.0	24.6 ± 4.0	37.6 (7.0)^c^
	4.5	55.6 ± 2.8	8.6 ± 1.5	35.6 ± 1.5	44.2 (3.0)^c^
	5.0	51.6 ± 20.1	5.0 ± 1.0	44.0 ± 18.7	49.0 (19.7)^c^
	5.5	2.3 ± 1.5	2.0 ± 1.0	95.3 ± 3.5	97.3 (4.5)^c^
	6.0	0.0	6.0 ± 4.5	93.6 ± 6.80	99.6 (11.3)^c^
	CTRL	99.3 ± 0.3	0.3 ± 0.5	0.3 ± 0.5	0.6 (1.0)
	NCL	0.0	0.0	100.0 ± 0.0	100.0 (0.0)
Gastrula	Potassium usnate				
	1.0	92.6 ± 3.7	6.0 ± 3.6	1.3 ± 1.5	7.3 (5.1)
	1.5	87.3 ± 6.1	3.6 ± 2.5	9.3 ± 3.2	12.6 (5.7)
	2.0	70.3 ± 6.4	19.0 ± 2.0^c^	10.6 ± 4.6	29.6 (6.6)^c^
	2.5	63.3 ± 4.3	15.3 ± 2.0^b^	24.6 ± 6.8^a^	39.9 (8.8)^c^
	3.0	51.5 ± 15.3	7.0 ± 8.7	41.6 ± 23.1	48.6 (31.8)^c^
	3.5	6.6 ± 2.0	3.6 ± 1.5	89.6 ± 1.5	93.2 (3.0)^c^
	4.0	0.0	0.0	100.0 ± 0.0	100.0 (0.0)^c^
	CTRL	98.6 ± 0.5	0.6 ± 0.5	0.6 ± 0.5	1.2 (1.0)
	NCL	0.0	0.0	100	100.0 (0.0)
Trocophore	Potassium usnate				
	1.0	96.0 ± .4.0	2.0 ± 1.0	1.0 ± 0.0	3.0 (1.0)
	1.5	92.6 ± 0.5	5.3 ± 1.5	2.0 ± 1.0	7.3 (2.5)
	2.0	83.0 ± 1.7	12.6 ± 4.0	2.3 ± 1.5	14.6 (15.5)^c^
	2.5	73.0 ± 5.0	23.6 ± 5.8	3.3 ± 2.3	26.9 (8.1)^c^
	3.0	67.3 ± 3.0	28.6 ± 2.5	4.0 ± 3.6	32.6 (6.1)^c^
	3.5	65.0 ± 2.0	22.3 ± 3.7	12.3 ± 3.5	34.6 (7.2)^c^
	4.0	48.0 ± 4.5	15.0 ± 7.9	37.3 ± 12.6	52.3 (20.5)^c^
	4.5	0.0	0.0	100	100.0 (0.0)^c^
	CTRL	99.0 ± 1.0	0.6 ± 0.5	0.3 ± 0.5	0.9 (1.0)
	NCL	0.0	0.0	100.0 ± 0.0	100.0 (0.0)
Veliger	Potassium usnate				
	1.0	97.6 ± 2.0	1.6 ± 0.5	1.0 ± 0.0	2.6 (0.5)
	1.5	83.0 ± 2.6	17.0 ± 1.7	0.33 ± 0.5	17.3 (2.2)^a^
	2.0	74.0 ± 6.0	18.3 ± 7.6	7.0 ± 5.0	25.3 (12.6)^c^
	2.5	74.0 ± 8.8	18.3 ± 8.9	7.6 ± 2.0	25.9 (10.9)^c^
	3.0	48.6 ± 12.1	26.3 ± 14.9	24.3 ± 2.5	50.6 (17.4)^c^
	3.5	27.6 ± 5.6	10.3 ± 5.7	62.3 ± 11.5	72.6 (17.2)^c^
	4.0	5.6 ± 2.5	5.6 ± 2.3	88.0 ± 4.5	93.6 (6.8)^c^
	4.5	0.0	4.3 ± 0.5	96.0 ± 1.0	99.6 (1.5)^c^
	CTRL	99.3 ± 0.5	0.3 ± 0.5	0.6 ± 0.5	0.9 (1.0)
	NCL	0.0	0.0	100.0 ± 0.0	100.0 (0.0)
Hippo stage	Potassium usnate				
	1.0	85.6 ± 1.5	0.6 ± 0.5	13.0 ± 3.6	13.6 (4.1)^a^
	1.5	69.6 ± 7.2	1.0 ± 1.0	28.0 ± 7.8	29.0 (8.8)^c^
	2.0	62.3 ± 2.5	0.6 ± 0.5	36.6 ± 3.0	37.2 (3.5)^c^
	2.5	53.3 ± 2.5	0.0	46.3 ± 0.5	46.3 (0.5)^c^
	3.0	46.0 ± 6.0	0.3 ± 0.5	53.3 ± 6.5	53.6 (7.0)^c^
	3.5	17.0 ± 2.6	0.0	82.6 ± 2.5	82.6 (2.5)^c^
	4.0	0.0	0.0	100.0 ± 0.0	100.0 (0.0)^c^
	CTRL	98.6 ± 0.5	0.6 ± 0.5	0.6 ± 0.5	1.2 (1.0)
	NCL	0.0	0.0	100.0 ± 0.0	100.0 (0.0)

Values expressed as mean (±standard deviation). Negative control group (CTRL) filtered and dechlorinated water only. Positive control group with 1.0 μg/mL niclosamide (NCL).

a The letters indicate significant differences *p* < 0.5 compared with the negative control (CTRL).

b The letters indicate significant differences *p* < 0.001 compared with the negative control (CTRL).

c The letters indicate significant differences *p* < 0.0001 compared with the negative control (CTRL).

**Table 2:** Lethal concentration (LC) for *S. mansoni* cercariae exposed to potassium usnate concentration 0.5, 1.0, 1.5, 2.5, 5, 10 and 100 µg/mL during 120 min.

**Exposure time (min)**	**Lethal concentration (µg/mL)**		
	**LC**_**10**_	**LC**_**50**_	**LC**_**90**_
**15**	1.59 [1.44–1,74]	4.22 [4.07–4,37]	8.38 [8.23–8.53]
**30**	1.07 [0.95–1.19]	3.29 [3.17–3.41]	5.98 [5.86–6.10]
**60**	0.59 [0.48–0.70]	1.98 [1.87–2.09]	4.93 [4.82–5.04]
**90**	0.31 [0.28–0.34]	1.16 [1.13–1.19]	3.37 [3.34–3.40]
**120**	0.19 [0.15–0.23]	0.71 [0.67–0.75]	2.41 [2.37–2.45]
**Niclosamide 1.0 µg/mL**	nc	nc	nc

nc=not calculated. [] 95% confidence interval.

## 2 Experimental design, materials and methods

### 2.1 *B. glabrata* embryotoxicity assay

The embryotoxicity assay was performed according to the methodology described by Araújo et al. [2]. *B. glabrata* embryos were collected by depositing polyethylene sheets (10 × 10 cm^2^) in aquarius. Subsequently, the embryos were packed in Petri dishes (10 mL) then, stereoscopic microscopes (Wild M3B, Heerbrugg, Switzerland) was used to evaluate and classify the embryos according to their stage of development following the methodology described by Kawano et al. [3]. The classification of the embryonic stages was determined after the first cleavage, as previously reported [4]: blastula (0–15 h), gastrula (24–39 h), trochophore (48–87 h), veliger (96–111 h) and hippo stage (144–168 h). Subsequently, groups of 100 embryos at each embryonic stage were exposed to 10 mL of potassium usnate in Petri dishes for 24 h at different concentrations as follows: blastula (1, 1.5, 2, 2.5, 3, 3.5, 4, 4.5, 5, 5.5 and 6 µg/mL); gastrula (1, 1.5, 2, 2.5, 3, 3.5 and 4 µg/mL); trocophore (1, 1.5, 2, 2.5, 3, 3.5, 4 and 4.5 µg/mL); veliger (1, 1.5, 2, 2.5, 3, 3.5, 4 and 4.5 µg/mL); and hippo stage (1, 1.5, 2, 2.5, 3, 3.5 and 4 µg/mL). Only filtered and dechlorinated water (pH 7.0) was used for the negative control (CTRL) groups. The positive group was prepared to consist of 1 μg/mL niclosamide (NCL) (Bayluscide, Bayer) in filtered and dechlorinated water. After 24 h of exposure, all embryos were washed with filtered and dechlorinated water and placed in new Petri dishes containing only filtered and dechlorinated water. After 8 days the embryos were analyzed daily and classified into viable (hatching) and unviable (malformed and dead). Two independent experiments were performed in triplicate.

### 2.2 Toxicity assay with *S. mansoni* cercariae

Cercariae of *S. mansoni* (Strain - BH) were obtained from of *B. glabrata* adults (*n* = 15) previously infected in a laboratory with miracidia (*n* = 6). After 35 days of infection, the snails were placed in a beaker of 400 mL and submerged in 100 mL of filtered and dechlorinated water and exposed to artificial light (60 W) for 2 h until the cercariae were eliminated to obtain the cercaria suspension. The assay was performed as described in a previous work [5], the estimation of cercariae was calculated by means of an inverted microscope (Leica DM IL Wetzlar, Germany) and an aliquot of 100 cercariae/mL was transferred to a concave glass container and exposed to solutions of the potassium usnate in final concentrations of 100, 10, 5.0, 2.5, 1.5, 1.0 and 0.5 μg/mL. Cercariae from the negative and positive control groups were exposed in filtered and dechlorinated water and to niclosamide at a concentration of 1 μg/mL, respectively. The cercariae were evaluated at intervals of 15, 30, 60, 90 and 120 min after exposure. The following parameters were used for the cercaricidal evaluation: mortality of 10% (LC_10_), 50% (LC_50_), and 90% (LC_90_) at different times after exposure. Two independent experiments were performed in triplicate.
