# Supplementary material for: Dataset on schistosomiasis control using potassium usnate against Biomphalaria glabrata at different developmental stage and Schistosoma mansoni cercariae
Source: Data Brief. 2018 Oct 27;21:1347–51. doi: 10.1016/j.dib.2018.10.119 (PMC6231030; doi:10.1016/j.dib.2018.10.119)
Supplement: Supplementary file 1 — Supplementary material. [file mmc1.pdf]

Conflicts of Interest Statement

Manuscript title: DataSet Schistosomiasis control using potassium usnate against *Biomphalaria glabrata* at different developmental stages and *Schistosoma mansoni* cercariae

The authors whose names are listed immediately below certify that they have NO affiliations with or involvement in any organization or entity with any financial interest (such as honoraria; educational grants; participation in speakers' bureaus; membership, employment, consultancies, stock ownership, or other equity interest; and expert testimony or patent-licensing arrangements), or non-financial interest (such as personal or professional relationships, affiliations, knowledge or beliefs) in the subject matter or materials discussed in this manuscript.

Author names: **Hallysson Douglas Andrade de Araújo**

Ana Maria Mendonça de Albuquerque Melo

Williams Nascimento de Siqueira

Mônica Cristina Barroso Martins

André de Lima Aires

Mônica Camelo Pessoa de Azevedo Albuquerque

Nicácio Henrique da Silva

Vera Lúcia de Menezes Lima

The authors whose names are listed immediately below report the following details of affiliation or involvement in an organization or entity with a financial or non-financial interest in the subject matter or materials discussed in this manuscript. Please specify the nature of the conflict on a separate sheet of paper if the space below is inadequate.

Author names:

This statement is signed by all the authors to indicate agreement that the above information is true and cor- rect (*a photocopy of this form may be used if there are more than 10 authors*):

Author's name (typed)	Author's signature	Date
Hallysson Douglas A. de Araújo	Hallysson Douglas Andrade de Araujo.	21/05/2018
Ana Maria Mendonça de A. Melo	Ana Maria M. de A. Melo	22/05/2018
Williams Nascimento de Siqueira	Williams Nascimento de Siqueira	21/05/2018
Mônica Cristina Barroso Martins	Mônica Cristina B. Martins.	22/05/2018
André de Lima Aires	André de Lima Aires	22/05/2018
Mônica Camelo P. A. Albuquerque	Mônica Albuquerque	22/05/2018
Nicácio Henrique da Silva	Nicácio Henrique da Silva	22/05/2018
Vera Lúcia de Menezes Lima	Vera Lúcia M. Lima.	22/05/2018
